# Which news topics drive economic prosperity in China?

**DOI:** 10.1371/journal.pone.0291862

**Published:** 2023-10-16

**Authors:** Wanbo Lu, Yifu Wang, Xingjian Zhang

**Affiliations:** 1 School of Management Science and Engineering, Southwestern University of Finance and Economics, Chengdu, Sichuan Province, China; 2 School of Statistics, Southwestern University of Finance and Economics, Chengdu, Sichuan Province, China; 3 Beijing Jingdong Technology Co., Ltd, Beijing, China; University of Barcelona: Universitat de Barcelona, SPAIN

## Abstract

Precise and real-time measurements of economic prosperity are vital to a country’s economic system. This study aims to identify news topics that promoted economic prosperity in China from 2011–2021. By extracting economic topics from news text data, we construct a news coincidence index with comprehensive information and strong timeliness and reveal the trend of topic contribution. The Latent Dirichlet Allocation (LDA) topic model is applied to extract economic topics from the news. We use a mixed-frequency dynamic factor model to track rapid economic development without using high-frequency weekly and daily data. We identify the six most influential topics and investigate their evolution, which may serve as a reference for economic construction and regulation.

## Introduction

Economic forecasts are crucial for modern economic research because they provide valuable insights for the government, businesses, residents, and other sectors. Improving accuracy is an ongoing challenge for economists. One reason for the often disappointing performance of economic forecasts is the delay in releasing indicators of economic activity, such as the Gross Domestic Product (GDP), which do not reflect real-time economic status [1, 2].

A promising strategy for addressing this challenge is using alternative datasets to complement the information provided by statistical agencies. These datasets are collected in real time from businesses and other sources, such as credit card transactions, online purchases, media tracking, and Internet searches. Aggregating detailed data from these sources can help produce indicators that can be incorporated into forecasting models. However, it is more reasonable for these alternative high-frequency indicators to complement instead of replace government agencies’ low-frequency macroeconomic data. Previous studies have shown that financial news and other types of text data contain important information about the future of the economy and can be used to explain economic fluctuations [3, 4]. Therefore, we construct a daily updated economic prosperity index based on news text data by extracting daily economic topics using LDA, which can provide inputs for real-time economic forecasts.

For this, we adopt a mixed-frequency dynamic factor model capable of bridging data with different frequencies that originated from the dynamic factor model [5]. Relevant studies on large mixed-frequency factor models include [6, 7]. Recently, [4, 8] have embodied economic news text data in a dynamic factor model. This study builds on the emerging literature that uses textual data from news to forecast economic variables, focusing on China. Although tokenization is considered more complicated in Chinese text analysis than in other languages, we believe that it allows us to track the economic cycle in a more real-time manner.

Several studies have examined the relationship between news texts and economic activity. For example, [9, 10] have forecasted economic growth using text data, whereas [11–13] have explored the association between newspaper texts and levels of uncertainty. The advantage of text-based indices lies in their predictive power [14, 15], timeliness [8, 16], and unique insights [15, 17]. One study particularly relevant to ours is [8]. In it, the author had constructed a daily business cycle index that incorporates text information using a time-varying factor model. However, unlike our study, [8] has used only one newspaper corpus and had a predetermined model specification. Additionally, they translated positive and negative words defined by the Harvard IV-4 Psychological Dictionary into Norwegian, which may have introduced bias. However, we adjust the tone of the news text using a raw Chinese sentiment dictionary. [15] have extracted timely economic signals from newspaper text and demonstrated that such information can significantly improve macroeconomic forecasts. However, they have not deeply delved into the information conveyed by the news text. Furthermore, all the studies mentioned have exclusively focused on English news texts, which differs from the focus of our study.

An economic prosperity index based on textual information is particularly interesting in the Chinese content for several reasons. China is the world’s second largest economy and a major player in international trade. Moreover, as an emerging economy, China has implemented various economic policy reforms that are reflected in the rich texts available. Therefore, an economic prosperity index based on textual information may provide important implications for China’s business cycles.

In this study, we present a novel approach to construct an intuitive economic prosperity index for China using 2.6 million news articles from three mainland Chinese news media covering the period from January 2011 to June 2021. The index covers a wide range of economic aspects in a timely manner and enables us to measure the contribution of each economic topic to the changes in economic prosperity. Our findings reveal that *Reform and Innovation*, *Infrastructure construction*, *Covid-19*, *Economic construction 2*, *International communication*, *Corporate innovation*, and *Culture and education* are the topics that contribute the most to China’s economic prosperity. Our study is part of the emerging literature that uses textual data from news to forecast economic variables, particularly in the US [9, 14, 18], the UK [15, 19], France [20], Germany [21], Italy [22], Norway [23], and Spain [24]. However, challenges remain regarding the selection of corpus, dictionary, and model specifications. For instance, [15] have demonstrated that existing positive and negative dictionaries differ in their forecasting performance. Therefore, we construct a news corpus containing official and emerging online news media utilizing a widely used Chinese sentiment dictionary. We also perform a robustness check using the dictionary, as proposed by [25].

Our most significant contribution is the proposal of a new approach that combines mixed-frequency dynamic factor models with Chinese text tokenization. Chinese text analysis is considered more complicated than that of other languages because of the lack of space between words. An algorithm combining a Directed Acyclic Graph (DAG) and Hidden Markov Model (HMM) is applied to extract words and phrases from sentences. This approach enables us to track the economic cycle in a more real-time manner despite the considerable computational complexity.

This study makes several contributions to existing literature. First, we generate a timely and interpretable economic index that captures important events and policies in China. The detailed illustrations of several important topic indices were provided. To the best of our knowledge, limited research in economics and finance has performed a textual analysis of news to measure daily economic development in China. Second, we use a hybrid source of news media, deriving data from both traditional news media and emerging Internet media to eliminate any selection bias in the corpus. Third, we implement the dynamic factor model under different restrictions, with or without tone adjustment, to strike a balance between model simplicity and accuracy. We offer a comprehensive comparison of the three different model structures and their performances with and without tone adjustment. Overall, our study provides new insights into the use of textual news data to measure daily economic development in China.

The remainder of this study is organized as follows. Section Methodology explains the construction of the index and measures topic contributions in detail. Section Empirical Analysis analyzes the dynamics of the index and economic topics. Finally, Section Conclusion concludes the study.

## Methodology

### The Latent Dirichlet Allocation topic model with Chinese texts

The LDA topic model is currently used in text mining for text topic recognition, text classification, and text similarity calculation. The LDA text topic model assumes that a text is written such that the writer first presupposes *K* topics for the text and then develops a sentence around *K* topics to express the text. The first step is to generate the initial proportional distribution of topics *P*(***θ***_***m***_; ***α***) in the document *m* by sampling from the Dirichlet distribution using the parameter ***α***. Second, the topic of the *n*th word in the document *m* is sampled from the multinomial distribution *P*(***Z***_*m*,*n*_|***θ***). Third, the initial word proportional distribution *P*(**Φ**_*k*_; ***β***) of topic *k* is generated by sampling from the Dirichlet distribution (using parameter ***β***). Fourth, the words ***W***_*m*,*n*_ are generated from the multinomial distribution of words $P(W_{m,n}|\Phi_{Z_{m,n}})$. Thus, the joint distribution of words and topics is as follows:

$$\begin{array}{c} P\left(W,Z,\theta ,\Phi ;\alpha ,\beta \right)=\prod_{k=1}^{K}P\left(\Phi_{k};\beta \right)\prod_{m=1}^{M}P\left(\theta_{m};\alpha \right)\prod_{n=1}^{N}P\left(Z_{m,n}|\theta_{m}\right)P\left(W_{m,n}|\Phi_{Z_{m,n}}\right). \end{array}$$ (1)

By integrating ***θ*** and **Φ** in Eq (1), we obtain:

$$\begin{array}{c} P\left(W,Z|\alpha ,\beta \right)=\int_{\theta }\int_{\Phi }P\left(W,Z,\theta ,\Phi ;\alpha ,\beta \right). \end{array}$$ (2)

**Fig 1:**
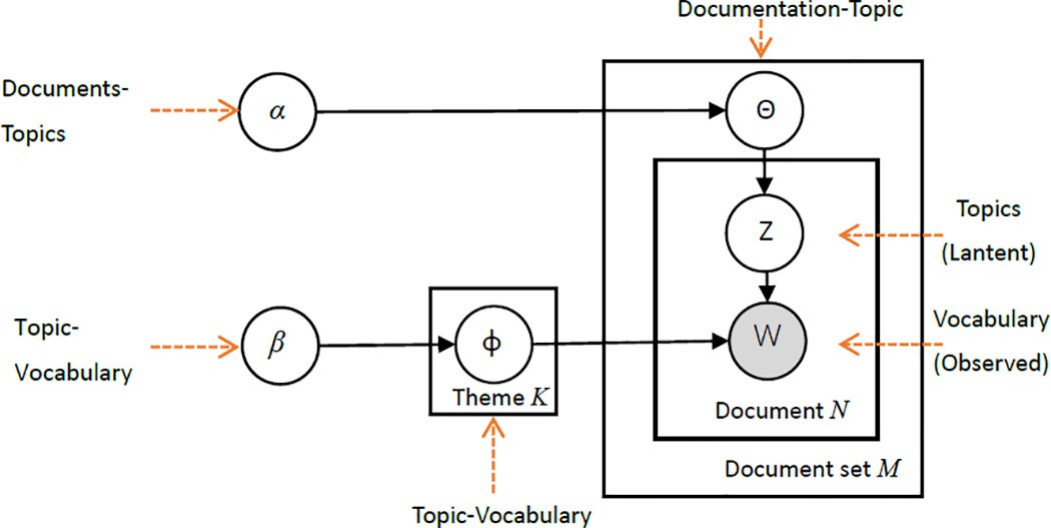
The plate notation for LDA.

Based on Eq (2), the parameters in the model are further estimated using Gibbs Sampling. The general concept of LDA is shown in Fig 1.

Specific nuances of Chinese text pose unique challenges that are different from languages using an alphabet, such as English, which require novel modifications to LDA in the tokenization step. The primary challenge is that Chinese characters do not have explicit boundaries. Therefore, there are multiple ways to segment a given piece of text into words, depending on the context and desired level of granularity. In this study, Jieba is applied to Chinese text segmentation. It uses a dictionary-based approach to segment Chinese text. It comes with a pre-built dictionary that includes over 300,000 words and phrases and uses an algorithm to identify the most likely segmentation of a given piece of text based on the frequency and co-occurrence of words in the dictionary.

### Mixed-frequency dynamic factor model and news coincidence index of economic prosperity

Since the publications of [26], economists have grappled with the question of how sentiment impacts economic decision-making. The relationship between sentiment data and decision making at the micro and macro levels remains a key area of interest in economic theory. Sentiment can be viewed as either containing fundamental information or as capturing irrationality and “animal spirits.” Both types of shocks affect economic expectations and market outcomes at different horizons [27]. However, according to [28], economists must first model and then quantify the sentiment forces behind market expectations. They have developed an economic theory that connects market expectations with market outcomes through external shocks, which is referred to as sentiments without departing from rationality. [29] have created a dynamic stochastic general equilibrium (DSGE) model that accommodates both the information and animal spirits views of confidence. They have found empirical evidence supporting the notion that innovations in confidence reflect information about future economic prospects. Economic sentiment is important because it can serve as a self-fulfilling prophecy [30]. When consumer or business pessimism about economic growth is widespread, negative growth can occur. Sentiment indices based on qualitative data can offer a more direct data-driven instrument for assessing various types of proxies, such as confidence or expectations. In this study, we incorporate the topics extracted by the LDA method into the dynamic factor model approach in [1] to estimate the common factors.

#### General settings

Let *x*_*t*_ denote the unobservable common factor (i.e., the extracted news coincidence index of economic prosperity) with a daily frequency. *x*_*t*_ follows the *AR*(*p*) process.

$$\begin{array}{c} x_{t}=\sum_{j=1}^{p}\rho_{j}x_{t-j}+e_{t}, \end{array}$$ (3)

where *e*_*t*_ is a white noise sequence with a mean 0 and a variance 1. Let *y*_*i*,*t*_ be the *i*th observable variable on day *t*, which, in this study, indicates the annual real GDP growth rates and economic topics. The expression of *y*_*i*,*t*_ is the same as in Eq (4)

$$\begin{array}{c} y_{it}=c_{i}+\beta_{i}x_{t}+\sum_{j=1}^{p}\gamma_{ij}y_{i,t-jD}+u_{it}, \end{array}$$ (4)

where *β*_*i*_ is the contribution of the news economic prosperity index to the *i*th observable variable and *y*_*i*,*t*−*jD*_ is the lagged term of *y*_*i*,*t*_. *D* is the number of days in each quarter of the corresponding year, with possible values of 90 (first quarter of leap years), 91 (first and second quarters of ordinary years), and 92 (third and fourth quarters). *p* is the number of lagged natural quarters and *γ*_*ij*_ indicates the correlation coefficient of *y*_*i*,*t*_ and *y*_*i*,*t*−*jD*_, which measures the persistence of *y*_*i*,*t*_. The *u*_*i*,*t*_s are not correlated, given that *t* and *u*_*i*,*t*_ are not correlated with *e*_*t*_.

Considering the lack of daily observations in low-frequency data, to perform operations on different frequency data in the mixed-frequency model, $y_{t}^{\prime }$ is denoted as the low-frequency expression of *y*_*t*_ (if there is a missing case in *y*_*t*_, it is also considered a low-frequency sequence and is denoted by $y_{t}^{\prime }$). The unobserved *y*_*t*_ is denoted by *NA*. If it is observable, then $y_{t}^{\prime }$ is the stock and keep *y*_*t*_ stays unchanged; however, if $y_{t}^{\prime }$ is the flow rate, the sequence of *y*_*t*_ is summed to the *p*th order lagged values. Using the state-space form, the model can be represented as follows:

$$\begin{array}{l} y_{t}=Z_{t}\alpha_{t}+\Gamma_{t}w_{t}+\varepsilon_{t},\\ \alpha_{t+1}=T_{t}\alpha_{t}+R\eta_{t}, \end{array}$$ (5)

where *ε*_*t*_ ∼ *N*(0, *H*_*t*_), *η*_*t*_ ∼ *N*(0, *Q*). *y*_*t*_ is the vector of the observable variable *y*_*it*_ consisting economic topics and real GDP growth rates. *α*_*t*_ is the state vector. *Z*_*t*_ is the factor loading matrix and Γ_*t*_ is the state transition matrix. *w*_*t*_ contains the constants and lagged terms of the observable variables. *ε*_*t*_ and *η*_*t*_ are the disturbances, while Γ_*t*_ and *R* are the coefficient matrices of *w*_*t*_ and *η*_*t*_.

#### Baseline mixed-frequency factor model for the news coincidence index of economic prosperity

The baseline mixed-frequency factor model must consider all the dynamic properties in the model. We construct the following model containing daily and quarterly indicators:

$$\begin{array}{c} \left\{ \begin{array}{c} y_{1,t}=\beta_{1}x_{t}+\mu_{1,t}\\ y_{2,t}=\beta_{2}x_{t}+\mu_{2,t}\\ \ldots \\ y_{30,t}=\beta_{30}x_{t}+\mu_{30,t}\\ y_{G,t}=\beta_{G}x_{t}+\gamma_{G}y_{G,t-Q}+\mu_{G,t} \end{array},\right. \end{array}$$ (6)

where *y*_1,*t*_, ⋯, *y*_30,*t*_ is the daily series of news topics extracted using the LDA model, *y*_*G*,*t*_ is the quarterly real GDP annual growth rate series, and *x*_*t*_ is the news coincidence index. *Q* is the daily interval in the natural quarter. *μ*_1,*t*_, ⋯, *μ*_30*t*,_ is a contemporaneous, uncorrelated series. In Eq (6), *γ*_*G*_ measures sustainability of *y*_*G*,*t*_. To make it easier for *x*_*t*_ to describe *y*_*G*,*t*_ while considering the dynamic nature of the model, the model form is streamlined by setting *γ*_*G*_ = 0, *ρ* = 1.

$$\begin{array}{c} \left\{ \begin{array}{c} x_{t}=x_{t-1}+e_{t}\\ \mu_{1,t}=\gamma_{1}\mu_{1,t-1}+\varepsilon_{1,t}\\ \ldots \\ \mu_{30,t}=\gamma_{30}\mu_{30,t-1}+\varepsilon_{30,t} \end{array}\right. \end{array}$$ (7)

We have the following measurement equation in the state–space model:

$$\begin{array}{c} \left[\begin{array}{c} y_{1,t}\\ y_{2,t}\\ \cdots \\ y_{29,t}\\ y_{30,t}\\ y_{G,t} \end{array}]=[\begin{array}{cccccc} \beta_{1} & 1 & 0 & \cdots & 0 & 0\\ \beta_{2} & 0 & 1 & \cdots & 0 & 0\\ \cdots & \cdots & \ldots & \cdots & \cdots & \cdots \\ \beta_{29} & 0 & 0 & \cdots & 1 & 0\\ \beta_{30} & 0 & 0 & \cdots & 0 & 1\\ \beta_{G} & 0 & 0 & \cdots & 0 & 0 \end{array}][\begin{array}{c} x_{t}\\ \mu_{1,t}\\ \mu_{2,t}\\ \cdots \\ \mu_{29,t}\\ \mu_{30,t} \end{array}]+[\begin{array}{c} 0\\ 0\\ 0\\ \ldots \\ 0\\ \gamma_{G} \end{array}]y_{G,t-Q}+[\begin{array}{c} 0\\ 0\\ 0\\ \cdots \\ 0\\ \mu_{G,t}, \end{array}\right] \end{array}$$ (8)

and the state equation,

$$\begin{array}{c} {}[\begin{array}{c} x_{t}\\ y_{1,t}\\ y_{2,t}\\ \cdots \\ y_{29,t}\\ y_{30,t} \end{array}]=[\begin{array}{ccccc} 1 & 0 & \cdots & 0 & 0\\ 0 & \gamma_{1} & \cdots & 0 & 0\\ \cdots & \ldots & \cdots & \cdots & \cdots \\ 0 & 0 & \cdots & 0 & 0\\ 0 & 0 & \cdots & \gamma_{29} & 0\\ 0 & 0 & \cdots & 0 & \gamma_{30} \end{array}][\begin{array}{c} x_{t-1}\\ \mu_{1,t-1}\\ \mu_{2,t-1}\\ \cdots \\ \mu_{29,t-1}\\ \mu_{30,t-1} \end{array}]+[\begin{array}{c} e_{t}\\ \varepsilon_{1,t}\\ \varepsilon_{2,t}\\ \cdots \\ \varepsilon_{29,t}\\ \varepsilon_{30,t} \end{array}]. \end{array}$$ (9)

#### News coincidence index of economic prosperity constrained by the disturbance lag

In the baseline model, the lag of disturbances in the daily frequency series is considered because of the missing daily data from some studies. Daily data are not missing in this study; therefore, to simplify the model in terms of parameters, the lag of the disturbance of the daily frequency series is eliminated, as compared to the baseline model. The resulting measurement equation can be expressed as follows:

$$\begin{array}{c} {}[\begin{array}{c} y_{1,t}\\ y_{2,t}\\ \cdots \\ y_{29,t}\\ y_{30,t}\\ y_{G,t} \end{array}]=[\begin{array}{c} \beta_{1}\\ \beta_{2}\\ \cdots \\ \beta_{29}\\ \beta_{30}\\ \beta_{G} \end{array}]x_{t}+[\begin{array}{c} 0\\ 0\\ 0\\ \ldots \\ 0\\ \gamma_{G} \end{array}]y_{G,t-1}+[\begin{array}{c} \mu_{1,t-1}\\ \mu_{2,t-1}\\ \cdots \\ \mu_{29,t-1}\\ \mu_{30,t-1}\\ \mu_{G,t} \end{array}], \end{array}$$ (10)

and the variance of the disturbances in the measurement equation is $H_{t}=[\begin{array}{cccc} \sigma_{1} & 0 & 0 & 0\\ 0 & \sigma_{2} & \cdots & 0\\ \cdots & \cdots & \cdots & \cdots \\ 0 & 0 & \cdots & \sigma_{G} \end{array}]$.

#### News coincidence index economy prosperity model constrained by the lag between disturbances and constant variances

A simplified model was used to set constant variance. The measurement equation is expressed as follows:

$$\begin{array}{c} {}[\begin{array}{c} y_{1,t}\\ y_{2,t}\\ \cdots \\ y_{29,t}\\ y_{30,t}\\ y_{G,t} \end{array}]=[\begin{array}{c} \beta_{1}\\ \beta_{2}\\ \cdots \\ \beta_{29}\\ \beta_{30}\\ \beta_{G} \end{array}]x_{t}+[\begin{array}{c} 0\\ 0\\ 0\\ \ldots \\ 0\\ \gamma_{G} \end{array}]y_{G,t-1}+[\begin{array}{c} \mu_{1,t-1}\\ \mu_{2,t-1}\\ \cdots \\ \mu_{29,t-1}\\ \mu_{30,t-1}\\ \mu_{G,t} \end{array}]. \end{array}$$ (11)

The state equation is expressed as follows: $H_{t}=[\begin{array}{cccc} 0.001 & 0 & 0 & 0\\ 0 & 0.001 & \cdots & 0\\ \cdots & \cdots & \cdots & \cdots \\ 0 & 0 & \cdots & 1 \end{array}]$.

### Decomposition of news coincidence index of economic prosperity via Kalman filter

In the state–space form of the mixed-frequency dynamic factor model, Eq (5), the *α*_*t*|*t*−1_ and *P*_*t*|*t*−1_ represent the predicted values of the state vector and its variance based on the information set at the time *t* − 1, respectively. *α*_*t*|*t*_ and *P*_*t*|*t*_ are the updated values of the state vector and its variance based on the information set at time *t*, respectively. The prediction formulae for Kalman filter are as follows:

$$\begin{array}{c} \alpha_{t|t-1}=T_{t}\alpha_{t|t},\\ P_{t|t-1}=T_{t}P_{t|t}T_{t}^{⊤}+RQR^{⊤}. \end{array}$$ (12)

Eq (12) are predicted using Eq (2), and later updated according to *y*_*t*_.

$$\begin{array}{c} \alpha_{t|t}=\alpha_{t}+P_{t}Z_{t}^{T}F_{t}^{-1}v_{t}\\ P_{t|t}=P_{t}-P_{t}Z_{t}^{T}F_{t}^{-1}Z_{t}P_{t}^{T}\\ v_{t}=y_{t}-Z_{t}\alpha_{t}-\Gamma_{t}\omega_{t}\\ F_{t}=Z_{t}P_{t}Z_{t}^{T}+H_{t} \end{array}$$ (13)

where *F*_*t*_ is the Kalman gain and *v*_*t*_ is the prediction error of the state vector. The maximum likelihood of the model is derived from the prediction error of the state vector.

$$\begin{array}{c} \text{log}\;L_{t}=-\frac{1}{2}\sum [N_{t}\;\text{log}\;2\pi +(\text{log}|F_{t}|+v_{t}^{⊤}F_{t}^{-1}v_{t})], \end{array}$$ (14)

where *N*_*t*_ denotes the dimensions of *y*_*t*_ at *t*. When calculating the maximum likelihood, if all elements *y*_*t*_ are missing, the contribution to the likelihood at *t* is 0. If a part of *y*_*t*_ can be observed at time *t*, then the contribution is $\left[N_{t}\;\text{log}\;2\pi +(\text{log}|F_{t}^{*}|+{v_{t}^{*}}^{⊤}{F_{t}^{*}}^{-1}v_{t}^{*})\right]$, where $F_{t}^{*}$ and $v_{t}^{*}$ are estimated using the Kalman filter.

## Empirical analysis

### Data

This study selects China’s economic news and related reports between January 1, 2011, and June 1, 2021. Considering that news with the same content can be reproduced many times, the news content in the economics section of each platform has high homogeneity. Therefore, obtaining news texts from all the mainstream news channels does not seem effective. The media platforms selected in this study are *People’s Daily*, *Guangming Daily*, and *Hexun*, which represent the news topics corresponding to the official, civil, and public sentiments, respectively. There are two main reasons for this selection. First, in terms of data representativeness, the *People’s Daily* was founded in 1946 and named as one of the top ten newspapers in the world by UNESCO in 1992. Its economic news section fully reflects the changes in topics related to the country’s economic policies. *Guangming Daily*, founded on June 16, 1949, is an ideological and cultural newspaper mainly aimed at intellectuals, covering more economic and cultural topics than *People’s Daily*. *Hexun*, founded in 1996, is China’s first financial information vertical website, and the only website in China that has the license to provide Internet information service, disseminate audiovisual programs on information network disseminations, and offer securities investment consulting, all at the same time. Its economic news content covers 25 subcategories in various fields, such as finance and securities, science and technology, and real estate, providing global 24X7 financial information to over 100 million annual users. This reflects the topics of concern and public sentiment in China’s private news coverage articles. Second, in terms of data accessibility, *People’s Daily* has established the *People’s Daily Graphic Database*, which uses electronic files to record all news text data since 1946. A part of the content is not accessible because reading permissions can be substituted by the content of *Guangming Daily*. We obtain financial news data from 2005 to the present by going through the scrolling news page of *Hexun*. We use the news headlines, instead of specific news contents, for the news published a long time.

A total of 260,000 articles are extracted from *People’s Daily* and *Guangming Daily*, accounting for only 2.96% of the total; whereas the number of *Hexun* articles exceeds 8.55 million, accounting for 97.04% of the total. This makes it easy for *Hexun* to occupy a dominant position when extracting topics. Potential text topics in the *People’s Daily* and *Guangming Daily*, which have relatively small volumes of news articles, cannot be reflected. Therefore, the text corpus of *Hexun* is intercepted. For every article, the headlines and news summaries are combined, instead of presenting the original text. The length of the text in the *People’s Daily* and *Guangming Daily* is 340 million words, accounting for 39.44% of the total, whereas the length of the text in *Hexun* is 530 million words, accounting for 60.56%. This represents a good balance given the number of articles. Basic information on the specific corpus is presented in Table 1.

**Table 1:** An overview of corpus.

Channels	Processing	No. of articles	% articles	Text length	% text length
People’s Daily and Guangming Daily	Complementary articles	260906	2.96%	343,818,582	39.44%
Hexun	Extract titles and abstracts	8550382	97.04%	527,880,382	60.56%
Overall	-	8811288	100.00%	871,698,964	100.00%

### News topic extraction

First, all news topics from the same day are collapsed into one row of data with two pre-defined Dirichlet distribution parameters ***α*** and ***β*** set to 1/*K*. It is found that there are a large number of topics with almost the same high-frequency words, which leads to insufficient differentiation among topics. In this situation, it is meaningless to extract topics. Decreasing *K* may result in a more compact and distinguishable group of topics at the expense of narrower coverage. Facing a tradeoff between the distinction and coverage of topics, this study conducts topic extraction experiments with *K* = 80, *K* = 70, *K* = 60, *K* = 50, *K* = 40, *K* = 30 and *K* = 20. A topic-word distribution with *K* = 30 is selected to strike a balance. After extraction, we find four groups of high-frequency words with Δ*GDP*. The correlation coefficients of the sequences are low, and the topics could not be extracted; therefore, they are eliminated. The final *K* = 26 results are listed in Table 2, where the correlation refers to the correlation coefficient between the topic and Δ*GDP*.

**Table 2:** Correlations of the topics and Δ*GDP*.

No.	Name	Correlation	No.	Name	Correlation
1	*Reform and Innovation*	0.0306	2	*Party building*	0.0029
3	*Infrastructure construction*	0.1064	4	*Social organisation structure*	-0.0018
5	*Traditional culture*	0.0075	6	*Legal system construction*	-0.0296
7	*Distinguished people*	0.0196	8	*Stock market*	-0.0004
9	*Olympic Games*	-0.0027	10	*News media*	0.0011
11	*Festive holidays*	0.0072	12	*Covid-19*	-0.4966
13	*Nature and ecology*	-0.0108	14	*Economic construction 1*	0.1524
15	*War abroad*	0.0158	16	*Chinese New Year*	0.0440
17	*Development and planning sessions*	0.0590	18	*Economic construction 2*	0.2043
19	*Geological disasters*	0.0146	20	*New Year*	0.0578
21	*International communication*	-0.1524	22	*the Two Sessions*	0.0761
23	*the National College Entrance Exam*	0.0603	24	*Corporate innovation*	-0.0051
25	*Culture and education*	0.1482	26	*Economic construction achievements*	0.0021

Correlation denotes the Pearson correlation coefficient between topic probability and Δ*GDP*.

The correlation coefficients of *Infrastructure construction*, *Covid-19* and *Economic construction 2* are higher. The correlation coefficient of the topic *Covid-19* with Δ*GDP* is -0.4966, which reflects the negative correlation between the pandemic and economic development. The correlation coefficients of *Economic construction 1*, *Economic construction 2*, and Δ*GDP* are 0.1524 and 0.2043, respectively. The correlation coefficient of *Culture and education* and Δ*GDP* is 0.1482. One aspect that needs to be clarified is that *Economic construction 1* and *Economic construction 2* spotlight the texts on economic plans and achievements from different viewpoints. The most important words in *Economic construction 1* are development, economics, business, construction, online, market, and investment. This topic focuses on development from the microeconomic perspective. The keywords are closely related to businesses and markets. Comparatively, the essential words in *Economic construction 2* are working sessions, economics, international, culture, conference, construction, and investment. This study focuses on development from a macroeconomic perspective. Accordingly, these keywords are closely related to policies and international trade.

Although different economic topic distribution sequences can be directly related to the Δ*GDP* sequence for establishong a relationship, the keywords of different topics mostly consist of nouns, adjectives, and degree adverbs that occur less frequently. Consequently, the same topic on different dates can have different effects on Δ*GDP* or even have an opposite effect caused by the different emotions expressed. Therefore, it is reasonable to adjust the tone of the distribution of each topic. In the study, we refer to the two-step procedure adopted by [4]. First, we collapse all newspaper articles for a particular day into one document and then compute the topic frequencies for this newly formed document using the estimated word distribution for each topic. This yields a set of K daily time series, in which each element represents how much (in percentage) a given topic is written about in a given day. Next, for each observation in the time series, we identify the signs (i.e., whether the news is positive or negative).

We use the China National Knowledge Infrastructure (CNKI) sentiment dictionary, a Chinese counterpart of the Harvard-IV dictionary, to construct positive, negative, and negation dictionaries to adjust the distribution of each topic. The CNKI sentiment dictionary is used for sentiment analysis in China. It is a collection of words and phrases that have been assigned sentiment scores based on their polarity (positive, negative, or neutral). The CNKI sentiment dictionary is widely used in China for sentiment analysis, and is considered a reliable resource for researchers and practitioners in the field. It is updated regularly to reflect changes in language usage and to improve their accuracy. An advantage of the CNKI sentiment dictionary is that it considers the unique linguistic and cultural context of Chinese-speaking communities. This makes it a valuable resource for sentiment analysis of Chinese texts because it can help overcome some of the challenges associated with sentiment analysis in this language. Although the CNKI sentiment dictionary, similar to any other sentiment dictionary, is not perfect and may have biases that affect the results of the sentiment analysis, it is representative because of the wide coverage of literature in its database.

Table 3 provides the descriptive statistics of the 26 themes before and after the sentiment adjustment, which represent the frequency of the topics over 10 years. The variances represent the stability of the topics. As shown in Table 3, topics with relatively high means and variances, such as *Culture and education*, *Reform and Innovation*, and *Infrastructure construction*, have a high probability of occurrence across all periods. The probability of the *Olympic Games* being held is high across all periods. The sentiment tendencies fluctuate even after sentiment adjustment. After sentiment adjustment, *Olympic Games*, *Covid-19*, *Economic construction 1*, *Development and planning sessions*, and *Geological disaster* have relatively large variances, indicating that the documents under these topics have emotional opposition and no clear emotional orientation. However, the other topics mostly maintain neutral sentiments. There are six other topics with negative mean values, due to the prevalence of more negative emotions, among which, *Reform and Innovation* and *Infrastructure construction* are the two topics of focus.

**Table 3:** Summary statistics of topics.

No.	Topic name	Before sentiment adjustment		After sentiment adjustment	
		Mean	St.d	Mean	St.d
1	*Reform and Innovation*	0.1493	0.2204	-0.0007	0.0076
2	*Party building*	0.0001	0.0044	0.0000	0.0000
3	*Infrastructure construction*	0.0880	0.1897	-0.0006	0.0056
4	*Social organisation structure*	0.0007	0.0200	0.0000	0.0000
5	*Traditional culture*	0.0000	0.0004	0.0000	0.0000
6	*Legal system construction*	0.0010	0.0182	0.0000	0.0001
7	*Distinguished people*	0.0001	0.0016	0.0000	0.0000
8	*Stock market*	0.0010	0.0159	0.0000	0.0003
9	*Olympic Games*	0.0134	0.0346	0.0000	0.0009
10	*News media*	0.0500	0.1301	0.0001	0.0027
11	*Festive holidays*	0.0209	0.0554	0.0001	0.0022
12	*Covid-19*	0.0777	0.1986	0.0000	0.0032
13	*Nature and ecology*	0.0001	0.0020	0.0000	0.0000
14	*Economic construction 1*	0.0242	0.0717	0.0000	0.0018
15	*War abroad*	0.0000	0.0006	0.0000	0.0001
16	*Chinese New Year*	0.0155	0.0568	0.0002	0.0023
17	*Development and planning sessions*	0.0101	0.0317	0.0000	0.0006
18	*Economic construction 2*	0.0735	0.1699	-0.0003	0.0053
19	*Geological disasters*	0.0040	0.0134	0.0000	0.0004
20	*New Year*	0.0214	0.0476	-0.0001	0.0016
21	*International communication*	0.0998	0.1821	0.0002	0.0046
22	*the Two Sessions*	0.0226	0.0652	0.0002	0.0022
23	*the National College Entrance Exam*	0.0264	0.0544	-0.0001	0.0018
24	*Corporate innovation*	0.0602	0.1337	-0.0002	0.0043
25	*Culture and education*	0.2356	0.2630	0.0013	0.0063
26	*Economic construction achievements*	0.0044	0.0097	0.0000	0.0002

Mean and St.d denote the mean and standard deviation of topic probability, respectively.

The six topics under investigation all exhibit negative sentiments for different reasons. Specifically, this analysis focuses on the following four topics: *Reform and Innovation*, *Infrastructure construction*, *the Chinese New Year*, and *the National College entrance examinations*(Gaokao). For these topics, negative sentiment scores are driven by negative comments and comparisons with other countries, such as the United States and Japan, which are mentioned in the corresponding topic’s keywords. Moreover, because of the complicated background of Reform and Innovation, which includes events such as the Opium War, it is difficult for algorithms to distinguish negative words within the narrative. Therefore, emotional adjustment is not considered in this analysis. Additionally, China’s anticorruption campaign efforts are highly debated, and the *Infrastructure construction* involves corruption and other related negative words contributing to the negative sentiment score.

For the remaining two topics, *the National College Entrance Exam* and *Corporate innovation*, the negative means are attributed to economic perspectives. According to [31], the Chinese national college entrance exam system faces the dilemma of adopting diverse and holistic college admissions, while preserving gaokao results as the sole admission criterion to ensure equal opportunities in higher education. Meanwhile, [32] have argued that a gender gap exists in the College entrance examination because of the lack of incentives to perform well in high-stakes settings. The corresponding tone-adjusted mean for these topics is negative because the discussion focuses more on achieving gender equality and diversification.

Corporate innovation and economic activities have a mixed relationship. [33] have found that economic policy uncertainty (EPU) is positively correlated with corporate innovation, suggesting that corporate innovation may induce potential risk. However, [34] have found that, as EPU increases, firms with more exposure to such uncertainty face a higher weighted average cost of capital and innovate less. The most relevant words for corporate innovation are Work, Innovation, Enterprise, Risk, Reform, and Investors. Negative emotions toward corporate innovation are rooted in media comments on the risks and uncertainties associated with this topic, which is reflected in the keywords containing risk and investors.

### Model selection and index construction

#### Economic prosperity index based on principal component analysis

**Fig 2:**
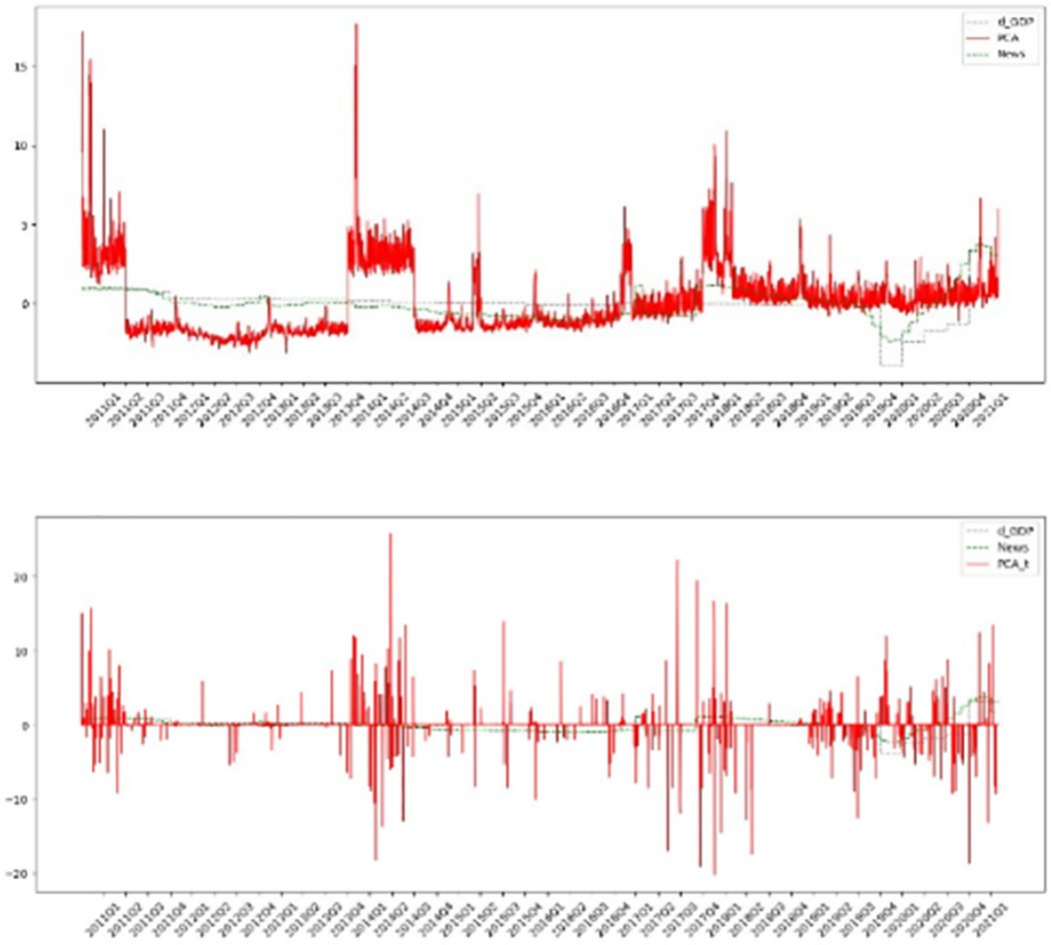
Comparing the first PC, prosperity index, and GDP growth rate. A (upper): the first PC (before sentiment adjustment), the CMPI, and GDP growth rate. B (lower): the first PC (after sentiment adjustment), the CMPI, and GDP growth rate.

Based on principal component analysis (PCA), the first principle component (PC) is extracted from the topic distribution before and after the sentiment adjustment. The real GDP yearly growth rate is calculated and the China Macroeconomic Prosperity index (CMPI) is compiled by National Bureau of Statistics of China. As shown in Fig 2, the PCA-based index is noisy because it fails to track the actual yearly GDP growth rate with different topic series. The RMSE of the index before sentiment adjustment, as compared to the real GDP yearly growth rate and the CMPI, are 2.26 and 2.04, respectively. Similarly, the RMSEs of the first principal component after sentiment adjustment, as compared to the real GDP yearly growth rate and the CMPI, are 2.25 and 2.27, respectively.

#### News coincidence index of economic prosperity based on dynamic mixed-frequency factor model

**Fig 3:**
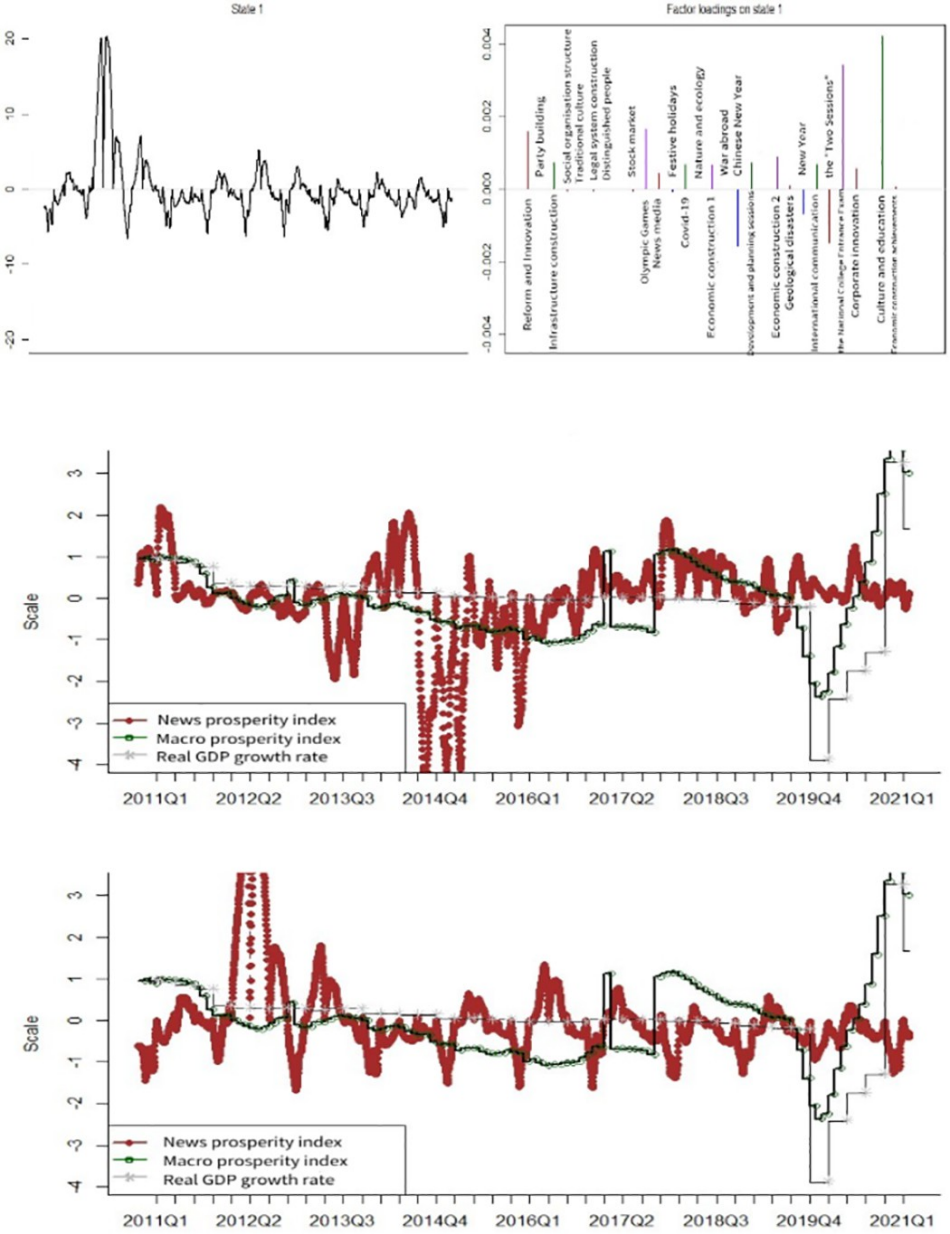
News topic loadings and index comparison for baseline model. A (upper): State 1 and the factor loadings on topics. B (middle): News prosperity index before sentiment adjustment. C (lower): News prosperity index after sentiment adjustment.

Fig 3 shows the news coincidence index of economic sentiment and news topic loadings in the benchmark model. The news topics with high news topic loadings are *Reform and Innovation*, *Infrastructure construction*, *the Olympic Games*, *Covid-19*, *Economic construction 2*, *the Spring Festival*, *International communication*, *the Two Sessions*, *Corporate innovation* and *Culture and education*. The benchmark model of the news coincidence index of economic prosperity is weak in reflecting the economic trends and turning points, identifying only the downturn between Q4 2011 and Q1 2012. The poor performance of the index in indicating and identifying economic prosperity, with or without adjusting for sentiment, is likely because of the complexity of the model structure, which results in some parameters not converging during iterations.

**Fig 4:**
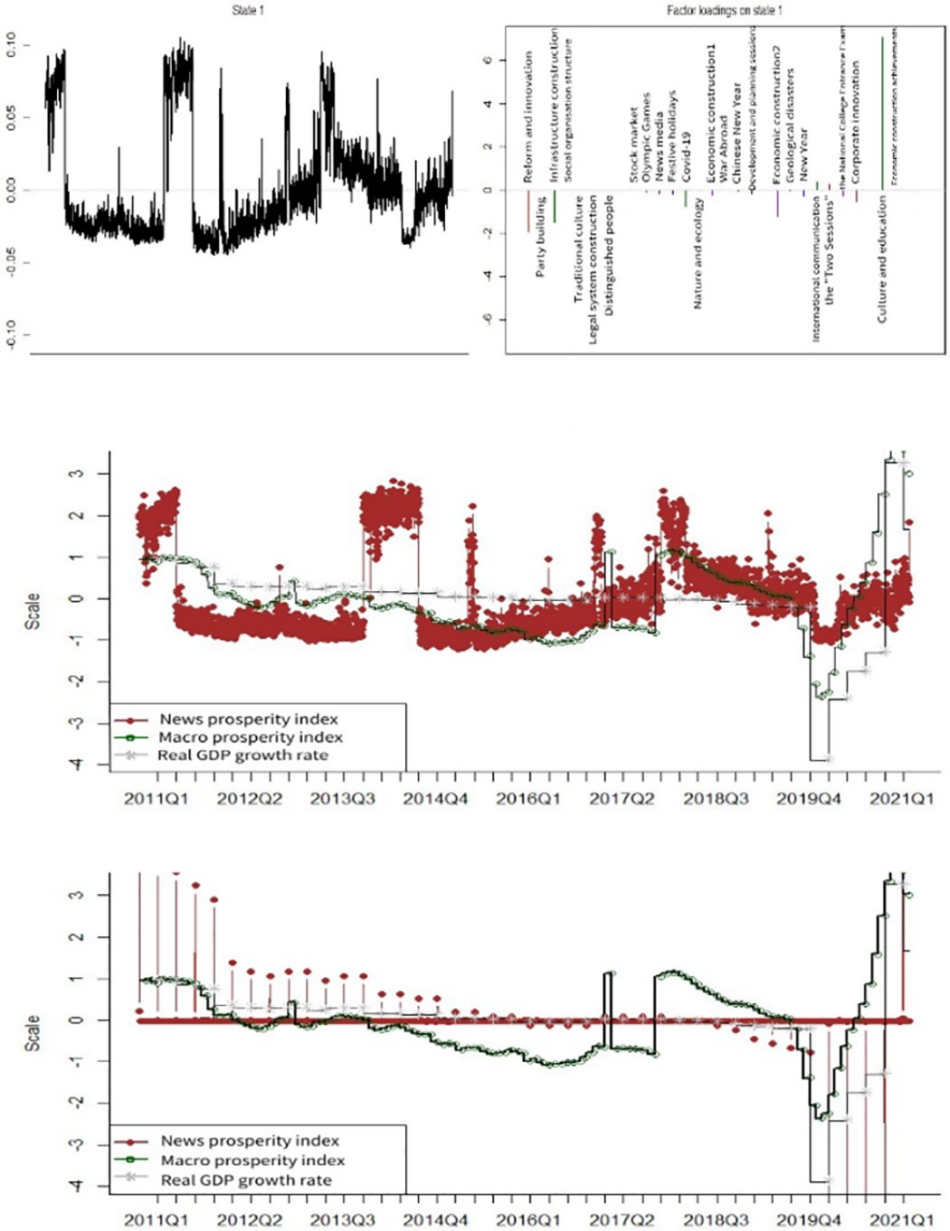
News topic loadings and index comparison for disturbance-restricted model. A (upper): State 1 and the factor loadings on topics. B (middle): News prosperity index before sentiment adjustment. C (lower): News prosperity index after sentiment adjustment.

Fig 4 illustrates the news coincidence index of economic sentiment and news topic loadings based on a model constrained by disturbance lag. Several news topics have higher news topic loadings in the form of restrictions on lagged disturbances; these include *Reform and Innovation*, *Infrastructure construction*, *Covid-19*, *Economic construction 2*, *International communication*, *the Two Sessions*, *Corporate innovation*, and *Culture and education*. At this point, the news coincidence index with the unadjusted sentiment can roughly describe the change in the macroeconomic index, including the prediction of the fourth quarter of 2011 to the first quarter of 2012 along with the economic downturns between the fourth quarter of 2016 and first quarter of 2017, and the fourth quarter of 2017 and the fourth quarter of 2018. In most other cases, the trend is consistent with that of the macroeconomic prosperity index and can determine the trend of large changes in advance. The results of the news sentiment index deviate from the macroeconomic prosperity index or the yearly growth rate of real GDP only from the fourth quarter of 2013 to the third quarter of 2014 and after the fourth quarter of 2019.

**Fig 5:**
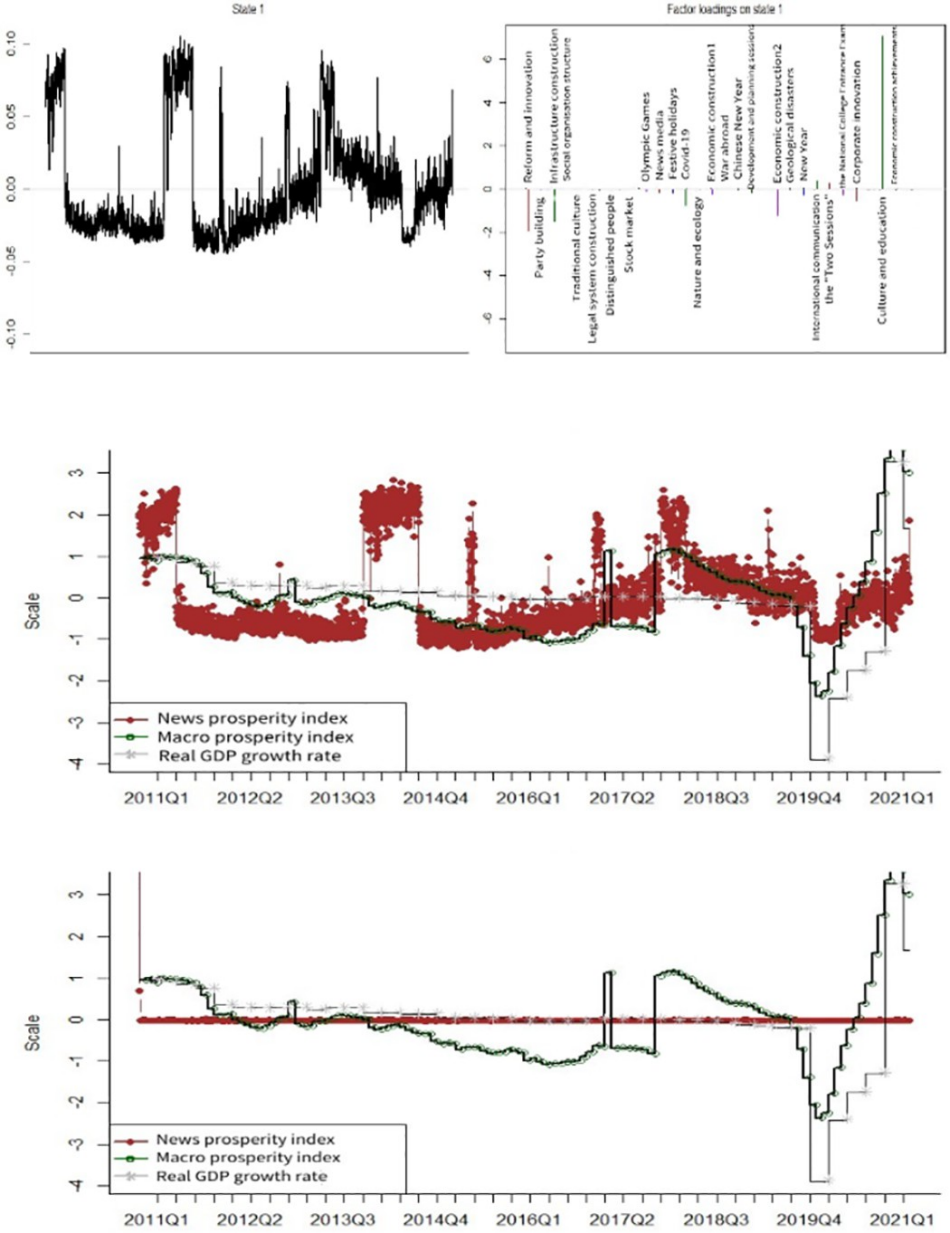
News topic loadings and index comparison for disturbance-restricted and constant variance model. A (upper): State 1 and the factor loadings on topics. B (middle): News prosperity index before sentiment adjustment. C (lower): News prosperity index after sentiment adjustment.

Fig 5 shows the news coincidence index of economic sentiment and news topic loadings in the model constrained by the lag of disturbances and constant variances. The number of news topics with higher news topic loadings with restrictions on lagged disturbances and constant variance is further reduced. Similar to the previous model, the trend of the news coincidence index in most cases is consistent with the trend of the macroeconomic prosperity index. Moreover, it can determine in advance the significant trend changes; however, from the fourth quarter of 2013 to third quarter of 2014, the results of the news coincidence index contradict the results of the macroeconomic prosperity index.

#### Comparative analysis and model selection

We compare the news coincidence indices of economic prosperity under the aforementioned three settings to determine whether is consistent with the macroeconomic prosperity index and whether it can reflect the turning point of the prosperity index in a timely manner. For this purpose, we consider the Akaike information criterion (AIC) with small sample correction and RMSE of model prediction. The results are listed in Table 4.

**Table 4:** Model comparison.

**Before sentiment adjustment**				
Model	(1)	(2)	(3)	(4)
Trend consistency	Basicly consistent	Basicly consistent	Most consistent	Most consistent
Turning point(s)	1	1	3	3
AIC		-292360	-4874220	-4904433
RMSE(CMPI)	2.04	1.47	1.17	1.18
RMSE(GDP)	2.26	1.38	1.30	1.30
**After sentiment adjustment**				
Model	(1)	(2)	(3)	(4)
Trend consistency	Basicly consistent	Basicly consistent	Consistent	None
Turning point(s)	2	2	1	None
AIC		-816697	-825770	-825539
RMSE(CMPI)	2.27	1.23	1.39	1.40
RMSE(GDP)	2.25	1.41	1.37	1.40

(1): Economic Prosperity Index based on PCA. (2): News coincidence index of economic prosperity based on baseline model. (3): News coincidence index of economic prosperity constrained by the disturbance lag. (4): News coincidence index economy prosperity model constrained by the lag between disturbances and constant variances

Considering the conditions before and after sentiment adjustment, the index trend consistency and RMSE before adjusting for sentiment are worse than those after adjusting the index for tone. However, the advantage of identifying turning points and AIC values is obvious, indicatng that tone adjustment smoothes the news sentiment consistency index. In the long term, the trend of the index after adjusting for tone is more consistent with that of the macroeconomic prosperity coincidence index. In the short run, non-adjusted data are more sensitive and accurate to the topic; hence, their prediction performance is better. With respect to the different models, the PCA-based index shows the weakest performance. With the simplification of the mixed-frequency dynamic factor model structure, the extracted news coincidence index of economic prosperity improves in terms of trend consistency and ability to identify turning points.

The complex structure of the benchmark model used to construct the news coincidence index may lead to deviations from the macroeconomic prosperity index and hinder its ability to identify turning points. Overfitting could be a reasonable explanation for this phenomenon because the nonzero coefficients of the lag term may not contribute to turning point identification. Conversely, the disturbance restriction model strikes a balance between complexity and accuracy, resulting in a more reliable news coincidence index. However, addition of a constant variance restriction to the disturbance restriction model does not improve the performance.

### Decomposition of news coincidence index of economic prosperity

Considering that the constant variance restriction has little effect on the actual constructed news coincidence index and is quite strong and subjective, the news coincidence index extracted by the disturbance restriction model without adjusting for sentiment is chosen for further analysis. Regardless of the model, *Reform and Innovation*, *Infrastructure construction*, *Covid-19*, *Economic construction 2*, *International communication*, and *Culture and education* are among the economic topics with the highest loadings. We document the changes in these six topics and how they reflect related policies.

**Fig 6:**
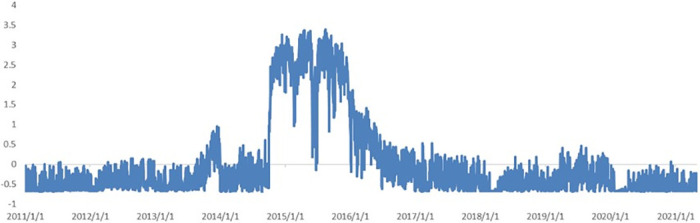
Dynamics of topic *Reform and Innovation*.

**Fig 7:**
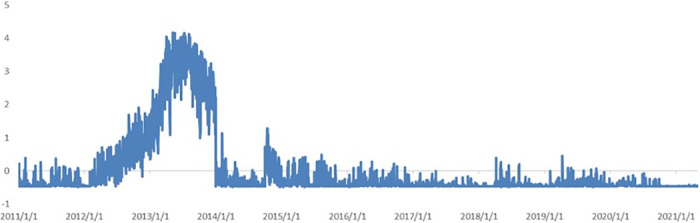
Dynamics of topic *Infrastructure construction*.

**Fig 8:**
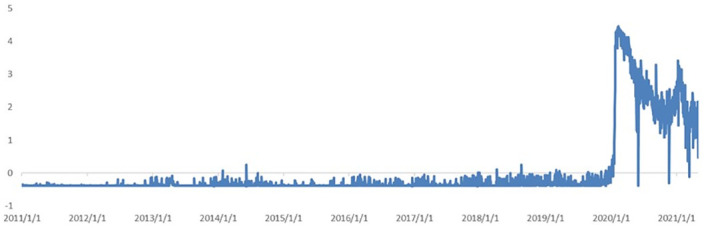
Dynamics of topic *Covid-19*.

**Fig 9:**
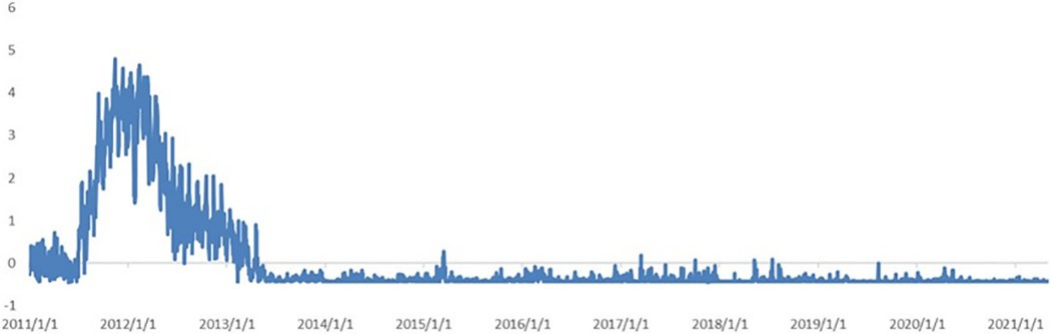
Dynamics of topic *Economic construction 2*.

**Fig 10:**
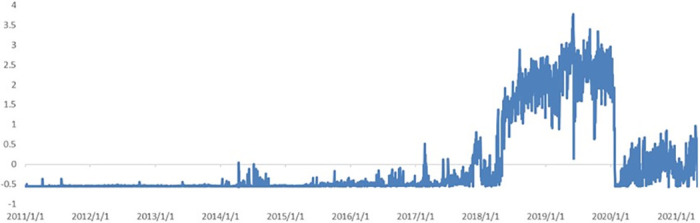
Dynamics of topic *International communication*.

**Fig 11:**
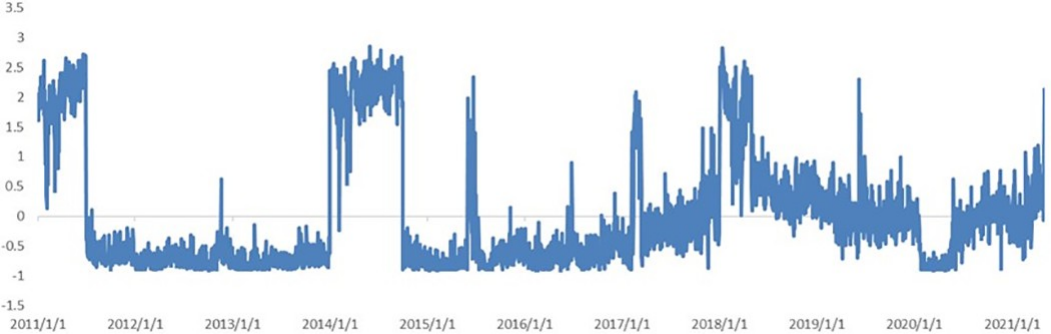
Dynamics of topic *Culture and education*.

(1) *Reform and Innovation*. As shown in Fig 6, the contribution of *Reform and Innovation* to the index increases slightly from 2013 to 2014, with the highest contribution being from 2015 to 2016. During this period, the deepening reform of the political system was comprehensively deployed, the reform of state-owned enterprises deepened, the implementation of the free trade zone strategy accelerated, and the “two-child” policy was implemented. These factors together sparked extensive discussions. In the context of increasing downward pressure on the economy and lack of a connection between old and new growth drivers, promoting entrepreneurship and innovation has been regarded as an important means of enhancing economic resilience and vitality. Accordingly, it has received unprecedented attention. The State Council has repeatedly emphasized that “double innovation” is the basic support for the economy to achieve medium-to-high-speed growth, and that “it should be relied on to stimulate the creative vitality of the whole society and create a new engine for economic development. In this context, China has witnessed a boom in entrepreneurship and innovation. The fact that the curve did not show significant growth or fluctuations after 2016 does not mean that the topic does not have affect the indicator, but that the influence of other indicators is too strong, resulting in a relatively small contribution of the topic.

(2) *Infrastructure construction*. Fig 7 shows the trend of the impact of *Infrastructure construction* on the news coincidence index from the first quarter of 2011 to first quarter of 2021. The trend in the curve in the figure represents the change in the topic’s contribution to the index. This contribution gradually increased from 2012 to the middle of 2013. In early 2014, it returned to its initial level, with only small fluctuations in early 2015. This period corresponds to the Twelfth Five-Year Plan period, when the country issued *the Medium and Long-Term Plan for Key National Technology Infrastructure Construction (2012–2030)*. This was a forward-looking plan and systematic deployment of major science and technology infrastructure construction to further improve the development level. It was important for enhancing China’s original innovation capability, achieving goals in key areas, guaranteeing the long-term development of science and technology, and achieving the goal of changing from a large science and technology-based country to a strong scientific and technological country. In March 2013, a series of incentives for infrastructure construction in the areas of livelihood, transportation, and cities allowed the high contribution of this topic to continue until the end of 2013.

(3) *Covid-19*. Fig 8 shows the trend of the impact of *Covid-19* on the news coincidence index. As shown in the figure, this topic has experienced explosive growth since the beginning of 2020. After pandemic prevention and control, the successful development of a preventive vaccine, and the gradual return to normal daily life, the contribution of *Covid-19* to the index gradually decreased and the curve dropped. However, with the arrival of winter, the pandemic again reached its peak transmission levels, and the contribution of the topic increased once more.

(4) *Economic construction 2*. Fig 9 illustrates the evolution of the impact of *Economic construction 2* on the news economy’s coincidence index. The period of 2011–2013 was the first half of the Twelfth Five-Year Plan, and the contribution of this topic began to significantly grow. It significantly increased until the end of 2012. The year 2012 was important for the implementation of the Twelfth Five-Year Plan. The State Council approved the 2012 key opinions on deepening the economic system reform.

(5) *International communication*. Fig 10 shows the trend in the impact of *International communication* on the news coincidence index. Since the beginning of 2018, the curve exhibited a more consistent upward trend. After former U.S. President Donald Trump signed a memorandum imposing punitive tariffs on Chinese products on March 22, 2018, China immediately announced its intention to impose tariffs on approximately 3 billion dollars of U.S. imports. This sparked widespread concern regarding US–China trade. During the same period, China’s *One Belt, One Road* construction project achieved milestones in international cooperation and project construction. A general connectivity framework consisting of six corridors, six connectivity routes, and multiple countries and ports was established. China built 82 overseas cooperation parks in the Belt and Road countries, paying more than 2 billion in taxes and fees to host countries and driving local employment to nearly 300,000 people. This provided more convenient living conditions, a better business environment, and more diverse development opportunities for people in these countries. After the outbreak of Covid-19 in early 2020, import and export businesses were greatly affected and international communications were reduced, but the curve gradually rebounded as China responded more quickly and various industries gradually recovered.

(6) *Culture and education*. As shown in Fig 11, the contribution of culture and education to the indicator has remained high, especially in 2011, 2014, and 2018. This is related to the full realization of the goal of “basically universalizing nine-year compulsory education and eradicating illiteracy among young adults” in the government work report released at the beginning of 2011. Additionally, the government has taken a series of initiatives to address the problems of “school choice fever” in compulsory education since 2014. These initiative addressed compulsory education in poor areas, special education, ideological and political education, student loans, and education in accordance with the law. Special governance actions were carried out in 2018 to curb the prominent problems brought about by the rapid development of out-of-school training institutions.

### Does dictionary matter?

This study employs universal sentiment dictionaries to analyze news texts predominantly written in the verbal form. As it can be argued that the universal sentiment dictionaries used in this study are more suitable for the oral context, we conducted a robustness check by replacing the China National Knowledge Infrastructure (CNKI) sentiment dictionary with the Chinese financial sentiment dictionary constructed by [25, 35]. [35] were the first to convert the English LM financial dictionary into a Chinese version. They then constructed a dictionary by screening out relevant emotional vocabulary from the Chinese General Emotional Dictionary and expanding the dictionary using the word2vec algorithm. However, we chose not to directly use this dictionary in our research because the translation of an English dictionary may introduce unnecessary bias.

Table 5 presents the topic distributions after dictionary substitution. First, we observe that the correlation coefficients of ΔGDP for the six major topics (*Reform and Innovation*, *Infrastructure construction*, *Covid-19*, *Economic construction 2*, *International communication*, and *Culture and education*) using two different dictionaries exhibit consistent signs. Second, the average topic scores are similar to those without tone adjustment after implementing the new dictionary. Furthermore, we calculate the RMSE of the prosperity index using a dynamic factor model with disturbance restrictions. The RMSE (GDP) and RMSE (CMPI) are 3.71 and 4.09, respectively, which are much larger than those reported in Table 4. This could be attributed to the fact that the new dictionary contains translations of the English text, which may introduce additional bias and may not be reliable. Therefore, we refrain from conducting further analysis of the dictionary. Overall, the main results are robust to the choice of the dictionary. However, some topics with mean values close to zero were sensitive to dictionary choices.

**Table 5:** Topic summary after sentiment adjustment by Chinese financial sentiment dictionary.

No.	Name	Mean	St.d	Correlation
1	*Reform and Innovation*	0.2144	0.2317	0.0411
2	*Party building*	0.0001	0.0046	0.0028
3	*Infrastructure construction*	0.0564	0.1357	0.0952
4	*Social organisation structure*	0.0007	0.0200	-0.0025
5	*Traditional culture*	0.0000	0.0003	0.0001
6	*Legal system construction*	0.0010	0.0185	-0.0286
7	*Distinguished people*	0.0001	0.0012	0.0277
8	*Stock market*	0.0000	0.0008	0.0192
9	*Olympic Games*	0.0000	0.0000	0.0254
10	*News media*	0.0988	0.2030	-0.0060
11	*Festive holidays*	0.0261	0.0666	0.0136
12	*Covid-19*	0.0776	0.2012	-0.5079
13	*Nature and ecology*	0.0003	0.0035	0.0626
14	*Economic construction 1*	0.0484	0.1301	0.2268
15	*War abroad*	0.0000	0.0000	0.0121
16	*Chinese New Year*	0.0002	0.0028	-0.0129
17	*Development and planning sessions*	0.0005	0.0043	0.0358
18	*Economic construction 2*	0.0189	0.0608	0.0537
19	*Geological disasters*	0.0000	0.0011	0.0139
20	*New Year*	0.0312	0.0853	0.0544
21	*International communication*	0.0680	0.1226	-0.1575
22	*the Two Sessions*	0.0048	0.0194	0.0401
23	*the National College Entrance Exam*	0.0392	0.0935	0.0560
24	*Corporate innovation*	0.0605	0.1240	0.1632
25	*Culture and education*	0.2528	0.2834	0.1266
26	*Economic construction achievements*	0.1266	0.0014	0.0266

Note: Mean and St.d denote the mean and standard deviation of topic probability, respectively. Correlation denotes the Pearson correlation coefficient between topic probability and Δ*GDP*.

## Conclusion

This study addresses three problems in current research on economic prosperity index, that is, the lag in publication, representativeness of index, and interpretability of index in the current research on macroeconomic prosperity index. We collect daily economic news text and quarterly real GDP yearly growth rate data to establish a connection between them using the LDA, mixed-frequency dynamic factor, Kalman filter, and other models. A news coincidence index of economic prosperity is constructed based on news topics that alleviate the aforementioned three problems. The following two main conclusions were drawn.

First, the text mining method can complement information ignored by the traditional economic prosperity index. Through empirical analysis, we find that the tone-adjusted index is better for fitting long-term macroeconomic trends, whereas the index without tone adjustment is better for short-term forecasts. With no missing daily data, the news coincidence index constructed using the benchmark model with a complex structure is more likely to deviate from the macroeconomic prosperity index. Thus, the ability to identify turning points is reduced. After a comprehensive comparison of trend judgment, the ability to identify turning points, AIC, and RMSE, the news coincidence sentiment index extracted by the disturbance-restricted model without adjusting sentiment data, is finally selected. It can describe the change in trend of the macroeconomic index and predict the economic downturn between the fourth quarter in 2011 and the first quarter in 2012. It can also explain the spikes in the prosperity index between the fourth quarter in 2016 and first quarter in 2017 and fourth quarter in 2017 and fourth quarter in 2018. The trend for most other cases is consistent with the trend of the macroeconomic prosperity index, which can predict the trend of large changes.

Second, we identified news topics closely related to economic prosperity. In this study, 26 topics were mined from news texts. In the correlation between topic probability distribution and GDP, *Infrastructure construction*, *Covid-19*, *Economic construction 2*, *International communication*, *the Two Sessions*, and *Culture and education* had a relatively high correlation with GDP, indicating that topic distribution can reflect changes in economic development. After decomposing the contribution of each economic topic into a news coincidence index, the following six topics were found to have a significantly higher impact: *Reform and Innovation*, *Infrastructure construction*, *Covid-19*, *Economic construction 2*, *International communication*, and *Culture and education*. We further investigated the impact of relevant events and policies on the evolutions of dominant topics.

Policymakers commonly consider a range of quantitative and qualitative information when making decisions regarding which statistical forecasts to use and how to measure sentiment and uncertainty. Using alternative datasets has the important advantage of allowing researchers to construct measures of economic activity on a daily basis and in real time, as is demonstrated by our sentiment indicators. This study has shown that text and text-based forecasts can complement policymakers’ decision-making by providing a timelier reading of economic activity, especially in situations with unavailable official.

Our empirical findings show that text mining methods can identify non-traditional economic topics. We decomposed the contribution of various economic topics to the news economic prosperity index and found that the following six topics had a significantly higher impact: *Reform and Innovation*, *Infrastructure construction*, *Covid-19*, *Economic construction 1*, *International communication*, and *Culture and education*. Note that *Covid-19* is not a common economic topic but can be extracted from news texts. Furthermore, the contributions of different news topics are distinct in different periods, and the evolution of topics is well reflected in the decomposition of the news coincidence index. For example, the contributions of topics such as *International communication* and *Covid-19* changed dramatically after 2020. However, further research is required to fully understand the potential and relevance of text analysis in different fields of economics and finance, including the construction of dictionaries specific to these fields. We also find that the model for constructing the economic prosperity index must be carefully specified to accommodate the frequency of macroeconomic data. We demonstrate that restrictions on the parameters can affect the quality of the index in terms of turning point identification and RMSE.

## Supporting information

S1 File (ZIP)
